# Assessment of Anti-Quorum Sensing Activity for Some Ornamental and Medicinal Plants Native to Egypt

**DOI:** 10.3797/scipharm.1204-26

**Published:** 2012-11-05

**Authors:** Ahmed A. Zaki, Mona I. Shaaban, Nadia E. Hashish, Mohamed A. Amer, Mohamed-Farid Lahloub

**Affiliations:** 1 Pharmacognosy department, Faculty of Pharmacy, Mansoura University, 35516, Egypt.; 2 Microbiology department, Faculty of Pharmacy, Mansoura University, 35516, Egypt.

**Keywords:** Antimicrobial agents, Antiquorum sensing, Herbal extracts

## Abstract

This study investigated the effects of some plant extracts on the bacterial communication system, expressed as quorum sensing (QS) activity. Quorum sensing has a directly proportional effect on the amount of certain compounds, such as pigments, produced by the bacteria. Alcohol extracts of 23 ornamental and medicinal plants were tested for anti-QS activity by the *Chromobacterium violaceum* assay using the agar cup diffusion method. The screening revealed the anti-QS activity of six plants; namely the leaves of *Adhatoda vasica* Nees*, Bauhinia purpurea* L.*, Lantana camara* L*., Myoporum laetum* G. Forst.; the fruits of *Piper longum* L.; and the aerial parts of *Taraxacum officinale* F.H. Wigg.

## Introduction

Antimicrobial agents exhibit their activity through different mechanisms such as disrupting cell wall function or disrupting protein and DNA synthesis [1–3]. As a result, multidrug resistance spreads rapidly, and development of new antimicrobial or antipathogenic agents that act upon new microbial targets becomes a very pressing priority [4]. Research efforts have focused recently upon developing antipathogenic agents to control bacterial diseases by inhibiting the communication between bacteria. Disturbing the bacterial communication system, or bacterial quorum sensing activity, causes attenuation of microbial pathogenicity [5–7]. Bacteria secrete specific extracellular signaling molecules called autoinducers, or acyl homoserine lactones (AHL), which are common in Gram-negative bacteria. The concentration of the autoinducers increases proportionally with the growth of a bacterial population and when it reaches a certain point, those signaling molecules diffuse back into the bacteria to regulate the transcription of specified genes. This regulation results in the control of many physiological processes such as: antibiotics production [8, 9], differentiation of a biofilm [10–12], cell division [13, 14], sporulation [14], secretion of virulence factors [15, 16], and primary metabolism regulation [17–19]. Thus, quorum sensing allows bacteria to control all essential processes, and could be considered as a promising and novel target for anti-pathogenic drugs, especially in combating bacterial infections caused by resistant strains. Developing new, non-toxic, and broad-spectrum anti-quorum sensing drugs from both microorganisms and plants is of great interest in recent years. Plants produce diverse antimicrobial compounds such as simple phenolics, catechins, quinones, flavanones, polyphenolics, alkaloids, and terpenoids [2, 20, 21]. Hence, bioscreening plant extracts for antiquorum sensing activity, followed by isolating the compounds responsible for this activity, is rational [22]. This study tested the anti-quorum sensing activity of some selected medicinal and ornamental plants, as a tool to biologically guide the isolation of the promising compounds. The plants used in this study are: *Adhatoda vasica* Nees, *Ambrosia psilostachya* DC., *Arctostaphylos uva-ursi* (L.) Spreng., *Bauhinia purpurea* L., *Boswellia carterii* Birdw., *Caesalpinia gillesii* Wall. ex Hook., *Chelidonium majus* L., *Dalbergia sisso* Roxb., *Datura stramonium* L., *Dimorphotheca ecklonis* DC., *Duranta erecta var. alba* (Mast.) Caro, *Kigelia pinnata* (Jacq.) DC., *Kochia indica* Wight., *Lantana camara* L., *Myoporum laetum* G. Forst., *Nerium oleander* L., *Olea europaea* L., *Piper longum* L., *Schinus molle* L., *Smilax aristolochiifolia* Mill., *Tagetes erecta* L., *Taraxacum officinale* F.H. Wigg., and *Zinnia elegans* Jacq. The anti-quourm sensing activities of these plants were evaluated using the QS biosensor *Chromobacterium violaceum* strain ATCC 12472.

To the best of our knowledge, studies to evaluate this activity for these selected plants have not been reported before.

## Results and Discussion

**Fig. 1:**
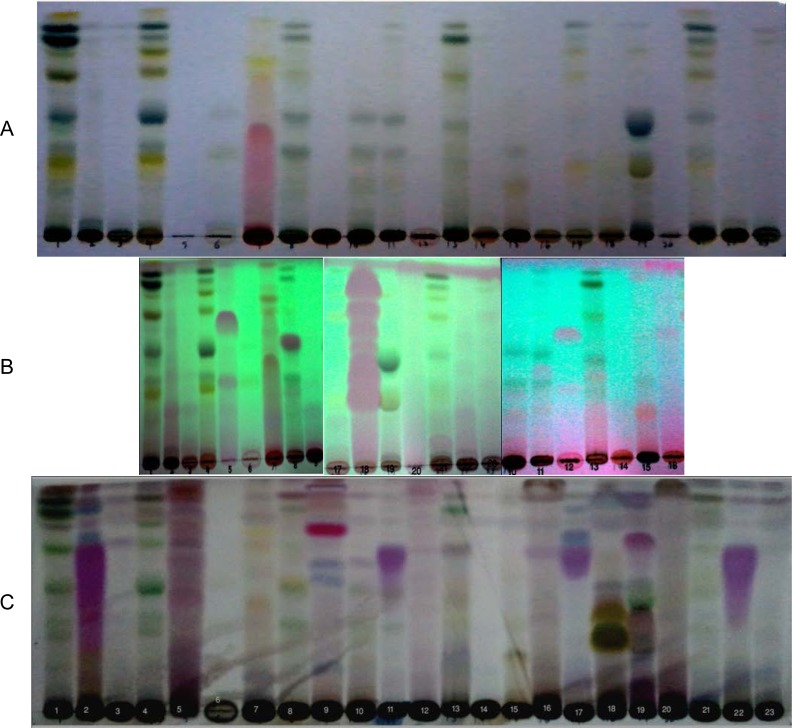
TLC profile of the total alcohol extracts of the plants under investigation A: Developed TLC, solvent system ethyl acetate:hexane (3:7); B: Visualization under UV 254 nm; C:TLC after spraying with Vanillin/ H_2_SO_4_ and heating at 110 °C for 5 min.

Phytochemical screening for the presence of alkaloids, saponins, carbohydrates, tannins, flavonoids, steroids, triterpenoids, and cardenolides is summurized in (Table 1). The thin-layer chromatography profile was carried out for the different extracts to compare them to each other (Fig. 1). The antipathogenic potential activities of plant extracts were evaluated by examining the antiquorum sensing activity of such extracts using the *Chromobacterium violaceum* assays. Pigment fading in the vicinity of the tested extracts indicated their QS inhibitory effect. Pigmentless zones adjacent to the clear zones of dead bacteria were observed surrounding certain samples, in comparison with the negative control, indicating the inhibition of violacein pigment secretion. Bacteria in this pimentless zones were alive but lost their QS ability.

The assay revealed, as shown in Table 2, that three plant extracts, namely the extracts of the leaves of *Myoporum laetum* G. Forst. *Adhatoda vasica* Nees and *Bauhinia purpurea* L., exhibited strong anti-quorum sensing activity/AHL-mediated violacein inhibition activities (15, 12, and 10 mm radius, respectively), while extracts of *Piper longum* L., *Taraxacum officinale* F.H. Wigg., and *Lantana camara* L. showed moderate anti-quorum sensing activity of 6–9 mm radius. Literature reviews for the anti-microbial activity of the total alcohol extracts of these plants revealed moderate to strongantimicrobial activities exhibited by the leaves of *Myoporum laetum* G. Forst [23], *Bauhinia purpurea* L. [24]*, Lantana camara* L. [25], *Taraxacum officinale* F.H. Wigg. [26]*, Adhatoda vasica* Nees**,** and the fruits of *Piper longum* L. The activity of the last two plants, *A. vasica* and *P. longum* L., was attributed to their alkaloidal content [27, 28].

## Conclusion

Six of the screened plants may contain antimicrobial compounds that we are currently working on isolating and identifying using the same bioassay as a guide. Thus, this study serves in selecting promising plant species for discovering new antimicrobial drugs.

## Experimental

### Plant material

Plants were collected either from the vicinity of Mansoura University or were purchased from commercial sources. Samples were identified by staff members in the Faculty of Agriculture. Voucher specimens were deposited in the herbarium of the Pharmacognosy Department, Faculty of Pharmacy, Mansoura University.

### Preparation of plant extracts

All plant samples were air-dried at room temprature and finely crushed. One hundred grams, dry weight, of each plant was extracted by soaking in 100ml 90% ethanol for two days with intermittent shaking; then the solvents were evaporated under reduced pressure on a rotary evaporator and the residues were stored frozen for testing.

### Phytochemical screening

Phytochemical screening was performed according to a standard procedure [29].

### Thin layer chromatography fingerprint

The extracts were run on pre-coated silica gel plates using a mixture of ethyl acetate:hexane (3:7) as the mobile phase. Spots were visualized by UV_254,365_ nm then sprayed with vanillin/sulfuric reagent and heated for 5 min. at 110 °C.

### Bacterial strains and growth conditions

#### Chromobacterium violaceum strain ATCC 12472

The strains were grown in Luria-Bertani (LB) broth (1% peptone, 0.5% yeast extract, 0.5% NaCl), solidified with 1.5% agar when required, and supplemented with an antibiotic (Kanamycin 20 μg/ml).

### Anti-quorum sensing assay

The quorum sensing inhibition activity of the plant extracts was determined by the agar cup diffusion assay, described by Zahin *et al*. [30] using the *Chromobacterium violaceum* strain ATCC 12472. In this test, bacterial growth inhibition would result in a clear halo around the cup, while a positive quorum sensing inhibition is exhibited by a turbid halo harboring pigmentless bacterial cells of the *C. violaceum* ATCC 12427 monitor strain. Cultures were prepared by growing bacteria in Luria Bertani broth (Merck, Germany) and incubated for 16–18 h in an orbital incubator (Labtech, Korea) running at 30 °C and 150 rpm. Cultures were then adjusted to 0.5 McFarland standard (Ca.10^8^ CFU/ml). Cups were of 10 mm diameter. Plant extracts were dissolved in sterile DMSO. A volume of 100 μl of each extract was transferred to the cups made in triplicate per extract onto *C. Violaceum*-inoculated (100 μl/plate) LB agar plates, which were then incubated at 30 °C for 24–48 h, and then the results were recorded (Table 2). The negative control was DMSO. Quorum sensing inhibition was calculated using the equation (r_2_− r_1_) in mm; where r_2_ is the total growth-inhibition zone radius and r_1_ is the clear zone radius [25].

## Figures and Tables

**Tab. 1:** Phytochemical analysis of total alcohol extracts of the studied plants

**#**	**Plant**	**Alk.**	**Sap.**	**Carb.**	**Tan.**	**Flav.**	**Ster.**	**Triterp.**	**Card.**
1	*Adhatoda vasica* Nees	+	–	+	+	+	+	–	–
2	*Ambrosia psilostachya* DC.	–	–	+	+	+	+	+	–
3	*Arctostaphylos uva-ursi (*L.*)* Spreng.	–	+	+	+	+	+	+	–
4	*Bauhinia purpurea* L.	+	+	+	+	+	+	+	–
5	*Boswellia carterii* Birdw.	–	–	+	–	–	+	+	–
6	*Caesalpinia gillesii* Wall. ex Hook.	+	–	+	+	+	+	+	–
7	*Chelidonium majus* L.	+	+	+	+	+	+	+	–
8	*Dalbergia sisso* Roxb.	–	–	+	+	+	–	+	–
9	*Datura stramonium* L.	+	+	–	+	–	–	+	–
10	*Dimorphotheca ecklonis* DC.	–	+	+	+	+	+	+	–
11	*Duranta erecta var. alba* (Mast.) Caro	–	+	+	–	+	–	+	–
12	*Kigelia pinnata* (Jacq.) DC.	–	–	+	–	+	+	–	–
13	*Lantana camara* L.	+	+	+	+	+	+	–	–
14	*Kochia indica* Wigh	+	+	+	+	+	+	+	–
15	*Myoporum laetum* G. Forst.	–		+	+	+	+	–	–
16	*Nerium oleander* L.	–	+	+	–	–	–	+	+
17	*Olea europaea* L.	–		+	+	+	+	+	–
18	*Piper longum* L.	+	–	–	+	–	+	+	–
19	*Schinus molle* L.	–		+	+	+	+	+	–
20	*Smilax aristolochiifolia* Mill.	–	+	+		+	+	+	–
21	*Tagetes erecta* L.	+		+	+	+	+	+	–
22	*Taraxacum officinale* F.H. Wigg.	–	+	+	+	+	+	+	–
23	*Zinnia elegans* Jacq.	–	+	+	–	+	+	–	–

Alk…Alkaloids; Sap…Saponins; Carb…Carbohydrates; Tan…Tannins; Flav…Flavonoids; Ster…Steroids; Triterp…Triterpenoids; Card…Cardenolides.

**Tab. 2:** Anti quorum sensing activity of the studied plant extracts against *Chromobacterium violaceum* strain ATCC 12472

**#**	**Plant**	**Parts used**	**Anti-QS zone^a^**
1	*Adhatoda vasica* Nees	Leaves	12±0.3
2	*Ambrosia psilostachya* DC.	Aerial parts	nil
3	*Arctostaphylos uva-ursi* (L.) Spreng.	Leaves	nil
4	*Bauhinia purpurea* L.	leaves	10±0.1
5	*Boswellia carterii* Birdw.	Oleoresin	nil
6	*Caesalpinia gillesii* Wall. ex Hook.	fruits	nil
7	*Chelidonium majus* L.	Aerial parts	nil
8	*Dalbergia sisso* Roxb.	leaves	nil
9	*Datura stramonium* L.	Seeds	nil
10	*Dimorphotheca ecklonis*DC.	Aerial parts	nil
11	*Duranta erecta var. alba* (Mast.) Caro	Leaves	nil
12	*Kigelia pinnata* (Jacq.) DC.	fruits	nil
13	*Lantana camara* L.	Leaves	9±0.6
14	Wight *Kochia indica*	Aerial parts	nil
15	*Myoporum laetum* G. Forst.	Leaves	15±0.4
16	*Nerium oleander* L.	roots	nil
17	*Olea europaea* L.	Leaves	nil
18	*Piper longum* L.	fruits	6±0.1
19	*Schinus molle* L.	Leaves	nil
20	*Smilax aristolochiifolia* Mill.	Roots	nil
21	*Tagetes erecta* L.	Flowers	nil
22	*Taraxacum officinale* F.H. Wigg.	Aerial parts	7±0.4
23	*Zinnia elegans* Jacq.	Flowers	nil

a mean of three readings (r_2_ − r_1_)

## References

[1] Arish D, Nair MS (2011). Synthesis, spectroscopic, antimicrobial, DNA binding and cleavage studies of some metal complexes involving symmetrical bidentate N, N donor Schiff base ligand. Spectrochim Acta A Mol Biomol Spectrosc.

[2] Cowan M (1999). Plant products as antimicrobial agents. Clin Microbiol Rev.

[3] Crémieux A, Chevalier J, Sharples D, Berny H, Galy A, Brouant P, Galy J, Barbe J (1995). Antimicrobial activity of 9-oxo and 9-thio acridines: Correlation with intercalation into DNA and effects on macromolecular biosynthesis. Res Microbiol.

[4] Coates A, Hu Y, Bax R, Page C (2002). The future challenges facing the development of a new antimicrobial drugs. Nat Rev Drug Discov.

[5] Finch R, Pritchard D, Bycroft B, Williams P, Stewart G (1998). Quorum sensing – A novel target for anti-infective therapy. J Antimicrob Chemother.

[6] Smith R, Iglewski B (2003). *Pseudomonas aeruginosa* quorum-sensing systems and virulence. Curr Opin Microbiol.

[7] Wu H, Song Z, Hentzer M, Andersen J, Molin S, Givskov M, Hoiby N (2004). Synthetic furanones inhibit quorum-sensing and enhance bacterial clearance in *Pseudomonas aeruginosa* lung infection in mice. J Antimicrob Chemother.

[8] Fineran P, Slater H, Everson L, Hughes K, Salmond G (2005). Biosynthesis of tripyrrole and β-lactam secondary metabolites in Serratia: integration of quorum sensing with multiple new regulatory components in the control of prodigiosin and carbapenem antibiotic production. Mol Microbiol.

[9] Slater H, Crow M, Everson L, Salmond G (2003). Phosphate availability regulates biosynthesis of two antibiotics, prodigiosin and carbapenem, in Serratia via both quorum-sensing-dependent and-independent pathways. Mol Microbiol.

[10] Coneye T (2010). Social interactions in the *Burhkolderia cepacia* complex: Biofilms and quorum sensing. Future Microbiol.

[11] De Araujo C, Balestrino D, Roth L, Charbonnel N, Forestier C (2010). Quorum sensing affects biofilm formation through lipopolysaccharide synthesis in *Klebsiella pneumonia*. Res Microbiol.

[12] Dickschat J (2010). Quorum sensing and bacterial biofilms. Nat Prod Rep.

[13] Chan Y, Chua L (2010). Growth-related changes in intercellular spermidine and its effect on efflux pump expression and quorum sensing in *Burkhodleria pseudomalei*. Microbiology.

[14] Jabbari S, Heap J, King J (2011). Mathematical modeling of sporulation-initiation network in Bacillus subtilis revealing the dual role of putative quorum sensing signal molecule PhrA. Bull Math Biol.

[15] Antunes L, Ferreira R, Buckner M, Finlay B (2010). Quorum sensing in bacterial virulence. Microbiology.

[16] Falcao J, Sharp F, Sperandio V (2004). Cell-to-cell signaling in intestinal pathogens. Curr Issues Intest Microbiol.

[17] Van Houdt R, Moons P, Hueso Buj M, Michiels C (2006). N-acyl-L-homoserine lactone quorum sensing controls butanediol fermentation in *Serratia plymuthica* RVH1 and *Serratia marcescens* MG1. J Bacteriol.

[18] Zhu H, Sun S (2008). Inhibition of bacterial quorum sensing-regulated behaviors by *Tremella fuciformis* extract. Curr Microbiol.

[19] Zhu H, He C-C, Chu QH (2011). Inhibition of quorum sensing in Chromobacterium violaceum by pigments extracted from *Auricularia auricular*. Lett Appl Microbiol.

[20] Adonizio A (2008). Anti-quorum sensing agents from South Florida medicinal plants and their attenuation of *Pseudomonas aeruginosa* pathogenicity. Ph.D. thesis.

[21] Dewick P (2002). Medicinal natural products: A biosynthetic approach.

[22] Bauer W, Tepletski M (2001). Can plants manipulate bacterial quorum sensing?. Aust J Plant Physiol.

[23] Earl EA (2010). Antibacterial effects of New Zealand plant extracts against mycobacteria.

[24] Murugan M, Mohan VR (2011). Evaluation of phytochemical analysis and antibacterial activity of *Bauhinia purpurea* L. and *Hiptage benghalensis* L. Kurz. J Appl Pharm Sci.

[25] Saraf A, Quereshi S, Sharma K, Khan NA (2011). Antimicrobial activity of *Lantana camara* L. J Exp Sci.

[26] Oseni LA, Yussif I (2012). Screening ethanolic and aqueous leaf extracts of *Taraxacum offinale* for *in vitro* bacteria growth inhibition. J Pharm Biomed Sci.

[27] Rangari PK, Patole VC, Chaudhari NA, Borhade PS, Devkar T (2012). Antimicrobial activity od *Adhatoda vasica* (Vasaca). Int J Pharmaschol.

[28] Trivedi MN, Khemani A, Vachhani UD, Shah CP, Santani DD (2011). Pharmacognostic, phytochemical analysis and antimicrobial activity of two Piper species. Pharmacie Globale.

[29] Trease GE, Evans WC (1989). Pharmacognosy: A physician guide to herbal medicine.

[30] Zahin M, Hasan S, Aqil F, Khan M, Husain F, Ahmad I (2010). Screening of certain medicinal plants from India for their anti-quorum sensing activity. Ind J Exp Bio.

